# Promises and pitfalls of electronic health record analysis

**DOI:** 10.1007/s00125-017-4518-6

**Published:** 2017-12-15

**Authors:** Ruth Farmer, Rohini Mathur, Krishnan Bhaskaran, Sophie V. Eastwood, Nish Chaturvedi, Liam Smeeth

**Affiliations:** 1 Department of Non-communicable Disease Epidemiology, London School of Hygiene and Tropical Medicine, Keppel Street, London, WC1E 7HT UK; 2 Institute for Cardiovascular Sciences, University College London, London, UK

**Keywords:** Diabetes, Electronic health records, Epidemiology, Observational studies, Primary care, Review, Secondary care

## Abstract

Routinely collected electronic health records (EHRs) are increasingly used for research. With their use comes the opportunity for large-scale, high-quality studies that can address questions not easily answered by randomised clinical trials or classical cohort studies involving bespoke data collection. However, the use of EHRs generates challenges in terms of ensuring methodological rigour, a potential problem when studying complex chronic diseases such as diabetes. This review describes the promises and potential of EHRs in the context of diabetes research and outlines key areas for caution with examples. We consider the difficulties in identifying and classifying diabetes patients, in distinguishing between prevalent and incident cases and in dealing with the complexities of diabetes progression and treatment. We also discuss the dangers of introducing time-related biases and describe the problems of inconsistent data recording, missing data and confounding. Throughout, we provide practical recommendations for good practice in conducting EHR studies and interpreting their results.

## Introduction

A greater understanding of the changing patterns of treatment, patient demographics, risk factors and disease burden is vital to inform clinical care and public health policy in diabetes. RCTs are key but will not answer all questions as they have several limitations: (1) they often have insufficient power and length of follow-up to examine clinical endpoints; (2) aspects of patient behaviour and clinical care are likely to differ in trials compared with real-world settings and (3) important groups, such as women of childbearing age, individuals with multimorbidities and ethnic minorities, may be under-represented in clinical trial populations [1–3]. On the other hand, classical cohort studies involving bespoke data collection are expensive and time consuming and rarely have long-term follow-up for participants beyond the initial study period.

The use of electronic health records (EHRs) for research allows us to overcome many of these limitations and address important scientific questions. Post marketing and surveillance studies using EHRs are key for speeding up access to new drugs [4]. Recognising this, the ADA recently endorsed the use of evidence from high-quality observational studies to aid therapeutic decision making [5, 6]. In recent years, the use of EHRs for research has grown tremendously and the potential for observational studies using EHRs to generate valid estimates of causal associations is beginning to be explored. Though EHRs have the potential to produce high-quality research, major challenges exist. In this narrative review, we describe the promises and potential of EHRs, outline some key areas of caution and provide practical recommendations for using EHRs in the context of diabetes research.

## The promise of EHR data

The term ‘electronic health record’ encompasses a wide variety of data sources including, but not limited to, routinely collected primary and secondary care records, disease-specific registries and health insurance claims databases (Table 1). Several key potential advantages of EHRs are outlined in the text box below.
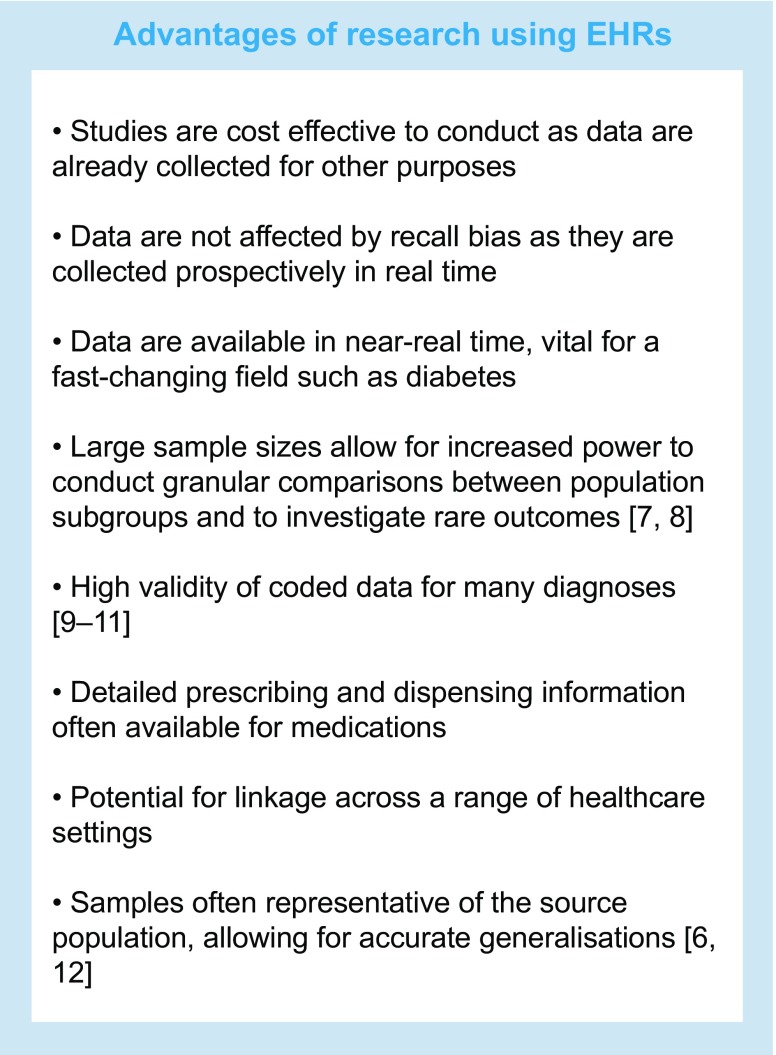

**Table 1:** Examples of EHRs

EHR	Data types available	Examples
Primary care databases	Diagnoses of chronic and acute conditions, prescription data, information on processes of care procedures and monitoring (e.g. blood tests, BP, screening and annual health checks), as well as demographic and lifestyle information such as age, sex, smoking and alcohol consumption	Clinical Practice Research Datalink (UK) QRESEARCH (UK) SAIL database (Wales) Primary Care Sentinel Surveillance Network (Canada) Integrated Primary Care Information Database (Netherlands) The Information System for the Development of Research in Primary Care (Spain)
Secondary care databases	Admissions to inpatient, outpatient and emergency services, diagnostic and procedural codes and administrative information such as length of stay, ward and specialty area	Hospital Episode Statistics (UK) National Registry of Patients (Denmark)
Disease registries	Detailed information on the relevant condition (e.g. cancer registries have details of date of diagnosis, cancer type, grade and treatments received but may lack information on comorbidities and concomitant medication)	Primary Care Cardiovascular Database (Sweden) Global Rare Diseases Patient Registry Data Repository (USA) Myocardial Ischaemia National Audit Project (UK) Danish Huntington Register (Denmark)
Insurance claims databases	Demographic information on the individual enrolled in the insurance plan, as well as details of medical history that have been covered and medication that has been claimed for under the insurance plan (e.g. information on prescription drugs and hospital inpatient and outpatient care)	Medicare (US) Health Maintenance Organizations (HMOs) such as Molina Healthcare, Kaiser Permanente, United Healthcare (USA) National Health Insurance Research Database (Taiwan) PHARM (Italy)
Pharmacy databases	Drug dispensing, effectiveness, safety and cost data	Scottish National Prescribing System (Scotland) PHARMO database (Netherlands) Deutsches Arzneiprüfungsinstitut (Germany)
Regulatory databases	Spontaneous reports of adverse drug reactions (ADRs)	Vigibase (WHO spontaneous reports database) EudraVigilance (Europe) GECEM (France)

EHRs are widely used to enable contemporary estimation of disease incidence or prevalence [13–15], study prospective associations between risk factors and disease outcomes [16], establish trends over time [17] and model the best use of healthcare resources [18, 19]. Importantly, many EHRs also provide high-quality data on medication prescribing. In claims databases, any medication claimed for under a health insurance policy is typically recorded by the insurance provider. In primary care databases, information on medications prescribed by the general practitioner (GP), such as number of tablets and dosage, are recorded, while in pharmacy databases, data on dispensing of medications are also available. Traditionally, data from EHRs have been used to assess adverse effects of treatment, especially unexpected effects. Improvements in the availability and quality of data and advances in study designs and analytical methods have broadened the value of such studies. This enables researchers to answer questions of both regulatory and epidemiological importance more quickly than with traditional study designs where data are collected in real time after conception of the study. EHRs have already been used to answer a range of questions concerning diabetes risk and treatment effects [20, 21].

Although no one database is likely to have a complete picture of an individual’s medical history, linkage between EHRs can improve completeness and validity of key morbidity data, as demonstrated for myocardial infarction [22], and enable the study of exposures and outcomes which would otherwise be impossible in unlinked data. In the UK, primary care data are routinely linked to Office for National Statistics death certificate data (providing detailed information on causes of death), hospital data (providing information on diagnoses from secondary care), deprivation data and disease-specific registries (e.g. for cancer and acute coronary syndromes) [12]. Similar linkages are also available between databases in the USA [23]. The availability of linked data depends greatly on the data provider, data infrastructure and, in the USA, healthcare provider. In Denmark and other Scandinavian countries, however, information across a wide range of databases (such as hospital records [11], prescriptions [24] and disease registries [25]) are all linked by a unique identity code assigned to each resident either at birth or when they become a resident [6], resulting in virtually complete population coverage and linkage. Linkages to biobanks can also provide highly detailed information on laboratory results and genetic markers (see for example http://www.bbmri-eric.eu/, accessed 5 June 2017); [26, 27]. Further, although different EHRs may use differing classifications and coding systems (e.g. Read codes vs ICD), combining data from multiple sources is still possible since mappings between coding and classification systems are generally available, or may be done on a study by study basis.

## Possible pitfalls of EHRs

We summarise a broad range of issues relevant to the study of diabetes using EHRs. A previous systematic review has detailed the methodological challenges of studying glucose-lowering medications in observational studies [28]. Therefore, issues specific to the study of drug effects, such as confounding by indication (whereby the reason for being prescribed [or not prescribed] the drug is itself related to the risk of the outcome), are not covered here.

### Accurate identification of diabetes status

Accurate disease ascertainment and categorisation is an essential first step towards identifying patterns of disease, and targeting interventions and resources appropriately. Challenges for diabetes researchers include the long latency between disease onset and diagnosis, and misclassification of diabetes type (e.g. older-onset type 1 diabetes being misclassified as type 2). Such misclassification may result in a biased estimation of the impact of diabetes on outcomes. Medication records may be used to supplement clinical data in identifying individuals with diabetes but this can present additional problems (e.g. metformin is used for the treatment of polycystic ovary syndrome and insulin is used in both type 1 and type 2 diabetes). Algorithms combining both diagnostic and supporting information (e.g. medication, laboratory results, age, BMI) have been developed to overcome these challenges [14, 29].

### Differentiating between prevalent vs incident disease and treatment

In many EHRs, individuals often join the database at time points with no clear clinical significance. For example, in primary care records, the first database entry is made on the date of an individual’s initial registration with the GP. At the initial visit, a GP may enter details for all pre-existing conditions. Therefore, in the period immediately after an individual enters the database, it may be unclear whether a new diabetes diagnostic code reflects existing diabetes or a new diagnosis [30]. This may limit the ability to adjust for diabetes duration, which may be an important source of confounding, particularly in studies comparing diabetes treatments. It is also typically unclear whether a new medication record in this early period reflects continuation of an existing therapy or incident use. Including prevalent users in a study of drug effects can lead to serious bias if treatment effects or risks vary over time, as is often (although not always) the case in diabetes. This is because prevalent users will have already ‘survived’ the early period of therapy [31]. For this reason, so-called new-user designs are generally encouraged, wherein new drug users are typically identified by requiring a certain period (e.g. 12 months) of follow-up before the first prescription [32]. However, it should be acknowledged that such designs may come at the price of loss in power, since we often reduce the sample to individuals with shorter exposure or duration of disease, which may reduce the number of long-term outcomes observed.

### Use of future information

When an EHR study is designed, it is often the case that all, or a large proportion, of the follow-up information is already available. Using future information when defining cohort inclusion, exposure status or covariate values at the time of study entry risks biasing the results because patient outcomes have influenced how they are dealt with in the study prior to their outcome [33]. As a simple example, consider a study of BMI and future risk of cardiovascular risk using a diabetes registry. Each individual may have multiple measures of BMI from the time they enter the registry until the time they exit the database or develop cardiovascular disease (CVD). If all BMI measures are used to determine whether an individual is overweight at study entry (e.g. by calculating an average BMI over follow-up), then the target comparison of ‘overweight’ vs ‘normal weight’ becomes a comparison of ‘average overweight’ vs ‘average normal weight’, leading to unclear interpretation and potential selection bias. An average normal weight could mask weight loss as a consequence of undiagnosed CVD, or a CVD diagnosis that appears late in the course of disease. Another problem of using future information is that concerning ‘immortal time bias’. This term is associated with the concept that during certain time periods during follow-up, a specific outcome cannot occur. Levesque et al [34] demonstrated this using data from a Canadian health database: they defined statin users as those with 12 or more months of continuous use during follow-up, and compared rates of insulin initiation (a proxy for diabetes progression) from study entry between users and non-users. This led to an estimated protective effect of statins. The problem with this approach is that anyone experiencing the outcome (insulin initiation) before completing 12 months of statin use would be classified as a non-user as their time at risk in the study would end at this point so they could not fulfil the definition of being a statin user. The corollary to this is that those categorised as statin users could not by definition have experienced the outcome (insulin initiation) prior to starting a statin and completing 12 months of statin use, creating a period of ‘immortal time’ for statin users. When this event-free person-time is included in the denominator, outcome rates in the exposed group are biased downwards, leading to an overall bias towards a protective effect of exposure. When the authors instead used a correct time-updated approach wherein an individual’s exposure status was updated from non-user to user once that individual reached 1 year from their first statin prescription, the protective effect of statins disappeared. Another solution might have been to start follow-up 1 year after the first statin prescription for statin users and to use a matched date for non-statin-users. Immortal time bias, along with other time-related biases, has been previously described in reference to studies of metformin and cancer risk in patients with diabetes [35] and in the previously referenced review by Patorno et al [28]. When defining inclusion criteria and exposures/covariates intended to reflect the point of study entry, it is worth asking the question ‘Have I only used information that I would have had at the time of recruitment had I conducted this study in real time?’ If the answer is no, then bias may inadvertently be introduced.

### Dealing with the complexities of diabetes progression

One of the most common scenarios in which bias from use of future information manifests in diabetes epidemiology is when dealing with treatment switches over the course of follow-up. Studies may restrict the study population to individuals who remain on a single therapy regime throughout follow-up, leading to selection bias or immortal time. One solution is to model the treatment of interest as time-varying, thus allowing the inclusion of all patients by accounting for their treatment modality. Such a solution would be relevant to the study of any exposure (e.g. BMI, HbA_1c_, eGFR) that changes as the disease progresses. Although an important advantage of EHRs is the ability to collect longitudinal data to investigate such time-varying exposures, dealing with confounding invariably becomes more complex in this situation. When considering how to model changes in exposure status through time, one must determine first whether information on time-varying confounders (confounders of the association between exposure and outcome that also change through time) is available in the database and second whether the time-varying confounders may also be affected by prior exposure status. If time-varying confounding is thought to be present, then adjustment for the value of the confounder at study entry only may not remove confounding for those whose exposure status changes over the course of follow-up. This can be overcome by using methods such as time-varying Cox proportional hazards models, which time-update the value of the confounder as it changes. However, if prior exposure is expected to affect future values of the confounder, then this method may not be appropriate as the adjustment may remove the effect of treatment that acts via future values of the confounder. These limitations of standard analysis methods in the presence of time-dependent confounders affected by prior exposures for diabetes research have been described in more detail in a systematic review [36], and more generally elsewhere [37]. Such issues occur both when examining time-varying treatment and time-varying risk factors such as BMI or glucose control or progressive conditions such as chronic kidney disease (CKD). For example, if we wish to examine the effect of CKD stage on mortality in individuals with diabetes, then HbA_1c_ may be a time-varying confounder of the association but CKD stage may also influence future HbA_1c_. Methodological approaches to dealing with time-varying confounders affected by prior treatment include inverse probability weighting of marginal structural models, g-computation and g-estimation [38]. In theory, these methods correctly adjust for the time-varying confounding without losing any effect of exposure that acts via future values of the confounder, subject to certain assumptions [38]. If such methodologies are not feasible, simpler study designs in which exposures are assumed to remain fixed from study entry (analogous to intention to treat analyses) may still be used to examine exposure/outcome associations but such designs can only answer more limited questions that ignore the reality of individuals changing treatments over time.

Finally, another consideration when dealing with time-varying exposure, is the extent to which changes in exposure are a result of reverse causality. For instance, many people lose weight shortly before diagnosis of diabetes, due to underlying ill health. Using weight measures shortly before diagnosis may lead to the erroneous conclusion that low weight is a risk factor for diabetes. It is advisable to conduct a sensitivity analysis to determine whether this may be an issue (e.g. by defining the date of exposure as being 6–12 months after the date observed within the EHR) [30].

### Context in which data are collected

Understanding the purpose for which the data were initially collected and methods of data collection are critical to accurate analysis and interpretation of EHR research and for assessing the likelihood of encountering problems of missing data and unmeasured confounding.

#### Selection biases arising from data availability

Primary and secondary care data are collected as and when individuals visit their GP or hospital and therefore samples from these databases may over-represent less-healthy individuals. This may present less of a problem in studies restricted to individuals with diabetes, since they will likely visit the GP on a semi-regular basis and thus have similar amounts and types of data recorded. However, if a general population comparison group is selected, those with available data may not be representative of the broader population. Even among individuals who do visit their GP regularly, there may be less data collected on those who are perceived to be healthier or at lower risk, as GPs are less likely to perform routine investigations in this group. Different considerations apply for claims databases: these may have an over-representation of healthier individuals, as those with pre-existing conditions may find it harder to receive medical cover.

#### Missing data

EHR data, for the reasons outlined above, likely suffer from missing data issues. Often, we classify variables based on the presence or absence of codes. For example, when determining whether an individual has had a previous CVD event, the presence of a code will indicate ‘yes’, while the absence of a code will likely indicate ‘no’, and thus we can derive a CVD status for 100% of individuals (albeit with the possibility of misclassification). However, for measures such as blood pressure or HbA_1c_, missing data are likely to indicate that the value has not been recorded. Analysing only the subset of individuals that have complete data on all necessary covariates is a commonly used approach but whether or not this is reasonable depends on how the missingness is associated with the outcome of interest [39]. Advanced methods such as multiple imputation may be used to assess the extent to which missing data may affect the analysis and to obtain more valid estimates of association if data are missing at random, meaning that the reason for missingness is independent of the value after conditioning on other measured covariates [40]. Unfortunately, this is an untestable assumption [40, 41] and often unlikely to hold. For example, smoking is more likely to be recorded in routine primary care among smokers, and BMI is more likely to be recorded among overweight individuals. Therefore, sensitivity analysis is always advisable and there exist comprehensive practical guides to approaching analysis with missing data [42, 43]. Even if observed, data on behaviours such as smoking and alcohol consumption are unlikely to be recorded with perfect accuracy, particularly since they are often self-reported and are subject to social desirability bias [44].

#### Unmeasured confounding

EHRs rarely contain information on diet and physical activity, which may be important confounders when looking at diabetes-related exposures and outcomes. Linkage to other sources may overcome this issue in some situations (e.g. some biobanks collect cross-sectional information on dietary intake). In some cases, proxies may allow some degree of adjustment for unobserved variables. For example, statin use may be a reasonable proxy for high cholesterol where actual cholesterol values are not recorded. If such options are not available, a negative control can be an informative way of investigating the impact of unmeasured confounding [45]. This involves examining an association that could plausibly be affected by the same unmeasured confounders as the primary association of interest, but where the true association is expected to be null. If the result obtained is close to the expected null association, this provides reassurance that unmeasured confounding is unlikely to be substantially biasing the results of the primary analysis. Such a method has been successfully employed by Jackson et al in debates over influenza vaccinations [46]. The authors estimated a protective association between vaccine use and trauma hospitalisation, suggesting that unmeasured confounding may be responsible for the observed reduction in respiratory hospitalisation.

## Recommendations

Although the challenges discussed in this paper were not identified systematically and were not intended to form an exhaustive list, they lead us to outline some key recommendations for best practice when studying diabetes using EHRs, as displayed in the following text box.
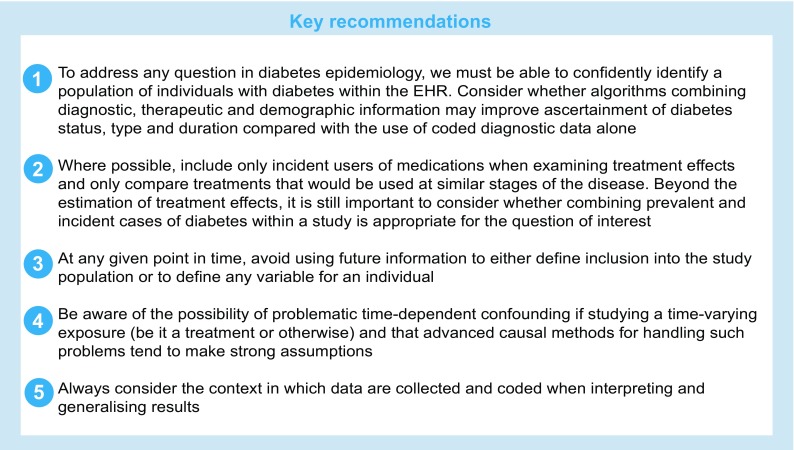


## Conclusions

EHRs offer great potential for the study of complex questions beyond the scope of traditional clinical and observational studies due to the breadth and timeliness of available data and the ability for linkage to secondary care, mortality data and disease registries. As such, there is a great opportunity to allow for more accurate characterisation of diabetes type, progression of disease and quality of care.

The increasing quantity and quality of computerised health-related data offers exciting opportunities for research in diabetes. However, the danger of poor quality research with misleading results is high and could result in deleterious effects on patient care and on prescribing. Improvements in reporting of research, driven by initiatives such as the Reporting of Studies Conducted using Observational Routinely Collected Health Data (RECORD) reporting guidelines statement, may make it easier to identify the most rigorous and reliable research [47]. Further, sharing of code lists and statistical code may improve reproducibility of research using EHRs. Alongside these improvements in transparent reporting, increasing awareness of the methodological challenges, such as those outlined in this paper, is needed to help ensure that studies based on EHR data produce valid results that usefully add to the evidence base.

## Abbreviations

CKD: Chronic kidney disease
CVD: Cardiovascular disease
EHR: Electronic health record
GP: General practitioner/general practice
